# Robotische Hernienchirurgie Teil IV: Roboterassistierte endoskopische parastomale Hernienversorgung. Videobericht und Ergebnisse

**DOI:** 10.1007/s00104-022-01715-7

**Published:** 2022-10-10

**Authors:** Maxime Dewulf, Ulrich A. Dietz, Agneta Montgomery, Eric M. Pauli, Matthew N. Marturano, Sullivan A. Ayuso, Vedra A. Augenstein, Jan R. Lambrecht, Gernot Köhler, Nicola Keller, Armin Wiegering, Filip Muysoms

**Affiliations:** 1 Department of Surgery, Maastricht University Medical Center, Maastricht, Niederlande; 2 Department of Visceral, Vascular and Thoracic Surgery, Cantonal Hospital Olten, Olten, Schweiz; 3 Department of Surgery, Skane University Hospital, Malmö, Schweden; 4 Department of Surgery, Division of Minimally Invasive & Bariatric, PennState Hershey Medical Center, Hershey, PA USA; 5 Division of Gastrointestinal and Minimally Invasive Surgery, Department of Surgery, Carolinas Medical Center, Charlotte, NC USA; 6 Department of Surgery, Sykehuset Innlandet Hospital Trust, Brumunddal, Norwegen; 7 Department of Surgery, Ordensklinikum Linz, Linz, Österreich; 8 Department of Urology, Cantonal Hospital St. Gallen, St. Gallen, Schweiz; 9 Department of General, Visceral, Transplant, Vascular and Pediatric Surgery, University Hospital Wuerzburg, Oberduer. Str. 6, 97080 Wuerzburg, Deutschland; 10 Department of Surgery, AZ Maria Middelares, Buitenring Sint-Denijs 30, 9000 Ghent, Belgien

**Keywords:** Parastomale Hernie, Ileum-Conduit, Pauli-Verfahren, Trichternetz (IPST), Modifizierte Sugarbaker-Technik, Parastomal hernia, Ileal conduit, Pauli procedure, Funnel mesh (IPST), Modified Sugarbaker technique

## Abstract

Die chirurgische Behandlung parastomaler Hernien gilt als komplex und ist bekanntermaßen komplikationsträchtig. In der Vergangenheit wurden diese Hernien durch die Relokation des Stomas oder Nahtverfahren der Austrittstelle versorgt. In den letzten Jahren wurden verschiedene netzbasierte Techniken vorgeschlagen, die heute in der minimal-invasiven Chirurgie eingesetzt werden. Mit der Verbreitung der roboterassistierten Hernienchirurgie wurden die Netzverfahren weiterentwickelt und die Ergebnisse für die Patienten erheblich verbessert. In diesem Beitrag wird ein Überblick über die verfügbaren Techniken der roboterassistierten Versorgung parastomaler Hernien präsentiert. Es werden technische Überlegungen und erste Ergebnisse des roboterassistierten modifizierten Sugarbaker-Verfahrens, der roboterassistierten Pauli-Technik und der Verwendung des trichterförmigen Netzes IPST vorgestellt. Darüber hinaus werden die Herausforderungen bei der roboterassistierten Versorgung parastomaler Hernien am Ileum-Conduit diskutiert. Die Operationstechniken werden durch Foto- und Videomaterial veranschaulicht.

**Video online:**

Die Onlineversion dieses Beitrags (10.1007/s00104-022-01715-7) enthält 4 Videos.

## Abstract (en)

The surgical treatment of parastomal hernias is considered complex and is known to be prone to complications. Traditionally, this condition was treated using relocation techniques or local suture repairs. Since then, several mesh-based techniques have been proposed and are nowadays used in minimally invasive surgery. Since the introduction of robot-assisted surgery to the field of abdominal wall surgery, several adaptations to these techniques have been made, which may significantly improve patient outcomes. In this contribution, we provide an overview of available techniques in robot-assisted parastomal hernia repair. Technical considerations and preliminary results of robot-assisted modified Sugarbaker repair, robot-assisted Pauli technique, and minimally invasive use of a funnel-shaped mesh in the treatment of parastomal hernias are presented. Furthermore, challenges in robot-assisted ileal conduit parastomal hernia repair are discussed. These techniques are illustrated by photographic and video material. Besides providing a comprehensive overview of robot-assisted parastomal hernia repair, this article focuses on the specific advantages of robot-assisted techniques in the treatment of this condition.

In dieser Übersichtsarbeit soll der aktuelle Stand der Technik der roboterassistierten Versorgung parastomaler Hernien vorgestellt werden. Die Autoren sind der Überzeugung, dass der roboterassistierte Ansatz, gestützt auf die teils ausgereifte Erfahrung mit Netzen und die jüngsten Verbesserungen der Kenntnisse über die Anatomie der Bauchwandschichten, eine vielversprechende Weiterentwicklung ist [1].

## Leben mit einem Stoma

Einer der prominentesten Chirurgen der Geschichte, der selber mit einem Stoma lebte, war der Norditaliener Edoardo Bassini (1844–1924), der Erfinder der ersten modernen Technik zur Reparation der Leistenhernie; Bassini wurde 1867 als Freiwilliger in der Armee von Giuseppe Garibaldi, die Rom zur Hauptstadt Italiens machen sollte, mit einem Bajonett verwundet. Infolgedessen entwickelte er eine Blinddarmfistel, zu seiner Zeit „Anus praeternaturalis“ genannt; es wird angenommen, dass er sein ganzes Leben lang als Chirurg mit diesem posttraumatischen Stoma lebte und arbeitete. Etwa 0,45 % der Bevölkerung in der westlichen Welt lebt mit einem Stoma, davon sind 60 % permanente Stomata [2]. Die häufigste Stomaart ist das Kolostoma (> 75 % aller angelegten Stomata).

Die problemlose Stomafunktion ist für gute Lebensqualität unerlässlich. Die Stomafunktion ist abhängig vom Stomatyp, der Stomaaustrittstelle an der Bauchdecke und der technischen Ausführung im Rahmen der Indexoperation. Es gibt drei validierte Instrumente, die zur Erfassung der Lebensqualität bei Stomapatienten verwendet werden: EORTC (European Organisation for Research and Treatment of Cancer) C30/CR38, MCOHQOLQO (modified City of Hope Quality of Life. Questionnaire Ostomy) und Stoma-QOL(Quality of Life)-Questionnaire [3].

Die häufigsten stomabezogenen Probleme sind die Versorgung mit dem Beutel, Hauterosionen, Schmerzen und die Entstehung einer parastomalen Hernie. In einer kürzlich durchgeführten Metaanalyse aus dem Jahr 2022, in der mehr als 1000 Patienten analysiert werden, lagen die stomabezogenen Komplikationen zwischen 3 und 80 % [4]. Wunde Haut und Ulzerationen sind mit einer Inzidenz von 25–35 % die häufigsten Komplikationen. Ein weiteres Problem ist die Entwicklung eines subkutanen Siphons des Stomas oder die Entwicklung einer „echten“ parastomalen Hernie, bei der Darmschlingen oder Omentum durch die Stomaöffnung neben der Stomaschlinge hernieren. Die Inzidenz der parastomalen Hernie wird auf etwa 30 % nach 12 Monaten, 40 % nach 2 Jahren und > 50 % bei längerer Nachbeobachtung geschätzt [5].

Technische Überlegungen bei der Anlage eines Stomas sind für die spätere Funktion des Stomas entscheidend.

## Parastomale Hernie

Die parastomale Hernie wird definiert als „eine abnormale Ausstülpung des Inhalts der Bauchhöhle durch die Öffnung der Bauchdecke, die bei der Anlage einer Kolostomie, Ileostomie oder eines Ileum conduits angelegt wurde“; dabei ist die Hernie vom Prolaps zu unterscheiden, bei dem der Darm teleskopartig – ohne die Notwendigkeit einer Hernie – durch das eigene Lumen evertierend prolabiert [6]. Die chirurgische Behandlung der parastomalen Hernie ist im Allgemeinen komplex und hat sich als komplikationsanfällig erwiesen [7, 8]. Da die lokale Behandlung dieser Hernien eine umfangreiche Dissektion der Stomaschlinge und die Implantation eines Netzes in unmittelbarer Nähe zum Dick- oder Dünndarm erfordert, zögern Chirurgen oft, diese Art von Operation durchzuführen.

Neben der Relokation des Stomas wurden verschiedene Techniken für die lokale Reparation parastomaler Hernien vorgeschlagen. In der aktuellen Literatur wird die Verwendung eines Netzes befürwortet, Nahtreparaturen werden hingegen als obsolet angesehen. Traditionell wurden diese Hernien mit einem Keyhole-Netz (bei dem die Stomaschlinge durch ein Loch im Netz verläuft) oder mit der modifizierten Sugarbaker-Technik (bei der die Stomaschlinge lateralisiert und ein intraperitoneales Netz zur Abdeckung der Austrittstelle implantiert wird) behandelt. Für die chirurgische Behandlung parastomaler Hernien wurden mehrere vorgeformte Netze untersucht, die meist intraperitoneal eingesetzt werden [9]. In jüngerer Zeit beschrieb Eric Pauli eine Modifikation der Sugarbaker-Technik, bei der die Stomaschlinge in der retromuskulären Ebene der Bauchdecke lateralisiert und ein extraperitoneales Netz verstärkend implantiert wird [10].

Aktuell gewinnt die roboterassistierte Bauchdeckenreparation rasant an Bedeutung. Für die Behandlung parastomaler Hernien bietet sie spezifische Vorteile, welche die Ergebnisse für die Patienten eventuell deutlich verbessern können:

- die Möglichkeit einer ausgedehnten Adhäsiolyse des Dünndarms von Treitz bis Bauhin,
- die Vermeidung schmerzhafter penetrierender Tacker, leicht ersetzbar durch Nahtfixation und
- ein Zugang zu neuen Bauchdeckenschichten, zum Beispiel über die posteriore Komponentenseparation in minimal-invasivem Ansatz.

In diesem Beitrag soll ein Überblick über die derzeit verfügbaren Robotertechniken bei der Versorgung parastomaler Hernien dargestellt werden, wobei der Schwerpunkt auf den operationstechnischen Überlegungen liegt. Außerdem werden vorläufige Ergebnisse dieser Techniken dargestellt. Dies ist der 4. Videobeitrag in einer speziellen Reihe zur roboterassistierten Hernienchirurgie in *Der Chirurg/Die Chirurgie* [11–13].

### Indikationen für die roboterassistierte parastomale Hernienversorgung

Künstliche Darmausgänge können unterschiedliche anatomische Merkmale aufweisen, welche einen Einfluss auf die Art der Reparation parastomaler Hernien haben: endständiges Kolostoma, doppelläufiges Kolostoma, endständiges Ileostoma (entweder als Enterostoma oder als Ileum-Conduit) oder doppelläufiges Ileostoma. Die Lage des Stomas kann in Abhängigkeit von der Topographie der muskuloskelettalen Strukturen variieren (z. B. transrektal, seitlich des Rektusmuskels, nahe oder weit entfernt von knöchernen Strukturen usw.). Die Qualität der Eversion des Darms auf Hautniveau kann ebenfalls Einfluss auf die Lebensqualität und Funktion eines Stomas haben. Im Allgemeinen wird die Indikation zur Versorgung einer parastomalen Hernie dann gestellt, wenn Pflegeprobleme der Haut bestehen, die Stomaplatte nicht gut haftet, der Patient Schmerzen hat oder Passagestörung auftreten. Auch Prolaps des Stomas mit oder ohne Schleimhautblutungen oder Polypen können eine Indikation für eine Reparation sein. Grundsätzlich gibt es keine guten technischen Möglichkeiten, die parastomale Hernie eines doppelläufigen Stomas zu versorgen, eine seltene Situation, welche die Behandlung von Patienten mit z. B. schweren Dysmotilitätsstörungen des Darmes erschweren kann.

Präoperativ wird eine Computertomographie (CT) des Abdomens und des Beckens angefertigt, welche Informationen über die Besonderheiten der Hernie gibt, wie z. B. den Durchmesser des Defekts, andere konkomitante Hernien (z. B. im Bereich einer ehemaligen Laparotomie), die Darmpassage, siphonartige Verwachsungen des Darms im Bruchsack und nicht zuletzt über zuvor implantierte Netze und Tacker bei Rezidivhernien. Computertomographische Bilder sind für die optimale Operationsplanung unerlässlich und helfen dem Chirurgen, wichtige Operationsschritte festzulegen, wie z. B. die Stelle für die Anlage des Pneumoperitoneums und den ersten Port zu definieren. Eine präoperative Koloskopie im Rahmen der Krebsvorsorge sollte in Betracht gezogen werden. Nicht zuletzt muss im Austausch mit den an der Behandlung des Patienten beteiligten Ärzten (z. B. Gastroenterologen, Kolorektalchirurgen und Urologen) evaluiert werden, ob das Stoma gegebenenfalls zurückverlegt werden kann. Wie auch bei Patienten mit anderen ventralen und inzisionalen Hernien, werden die beeinflussbaren perioperativen Risikofaktoren vor der Operation optimiert [14, 15]. Im Allgemeinen wird ein Body-Mass-Index (BMI) von weniger als 35 kg/m^2^ angestrebt.

### Operationsvorbereitung und Lagerung

Der Eingriff erfolgt in Vollnarkose, der Patient wird in Rückenlage gelagert. Je nach Stomaposition, wird der Patient rechts- oder linksbündig auf dem Operationstisch positioniert: Der Rumpf des Patienten wird zur Seite, wo die Ports positioniert werden, bündig an die Tischkante gelagert und der Patientenarm wird leicht abgewinkelt etwas unterhalb der Tischhöhe angelagert, um die Bewegungsfreiheit der Roboterarme zu optimieren. Der Tisch kann dorsal flektiert werden, was die Distanz zwischen Rippenbogenrand und Spina iliaca anterior superior erweitert und einen größeren Arbeitsbereich für die Roboterarme ermöglicht. Wenn ein beidseitiger Transversus-abdominis-Release (TAR) erforderlich ist, erfolgt der Zugang von beiden Seiten und der Patient kann mittig gelagert werden. Wegen der möglichen Operationsdauer ist ein Harnkatheter empfohlen, falls es sich um ein Ileum-Conduit handelt, wird dieses mit einem sterilen Foley-Katheter sondiert, um Urinstau zu verhindern und im Rahmen der peristomalen Adhäsiolyse die Stomamorphologie besser darstellen zu können oder ggf. sogar mittels eines Einlaufs mit Blaulösung Deserosierungen auszuschließen. Die Stomaöffnung wird bei geplanter In-situ-Relokation, mit einer Naht verschlossen, ansonsten reicht meist ein Okklusivverband mit Klebefolie, um das Auslaufen von Flüssigkeit zu minimieren und die Kontamination der Wunde zu verhindern. Prophylaktische Antibiotika sollen entsprechend den lokalen Krankenhausprotokollen verabreicht werden. Der DaVinci Xi (Intuitive Surgical, Sunnyvale, CA) mit Doppelkonsole und Tischbewegung (Trumpf Medical, Deutschland) ist die bevorzugte Option.

Unabhängig von der Art der roboterassistierten Reparation der parastomalen Hernie ist die Positionierung der Ports ein Kompromiss zwischen optimalem Abstand zur Bruchstelle und den angrenzenden knöchernen Strukturen, unter Berücksichtigung konkomitanter Hernien und die Wahl eines Bereichs des Abdomens, wo Verwachsungen weniger wahrscheinlich sind. Häufig ist eine Adhäsiolyse erforderlich, eventuelle Deserosierungen müssen zeitnah erkannt und übernäht werden. Wenn der Operationsablauf beonders schwierig oder der Patient instabil ist, kann der Eingriff auf hybride Weise fortgeführt werden (z. B. offene peristomale Adhäsiolyse), in seltenen Fällen muss konvertiert werden. Wenn Bedenken hinsichtlich der Blutversorgung des Mesenteriums bestehen, werden 0,2 mg/kg pro 3 ml (oder vereinfacht 1 ml einer 5 mg/ml Suspension) Indocyaningrün (ICG) intravenös verabreicht, die robotergestützte Firefly-Technologie erlaubt in Echtzeit die Beurteilung der Perfusion des Gewebes [16].

## Modifizierte Sugarbaker-Technik

**Figure 1:**
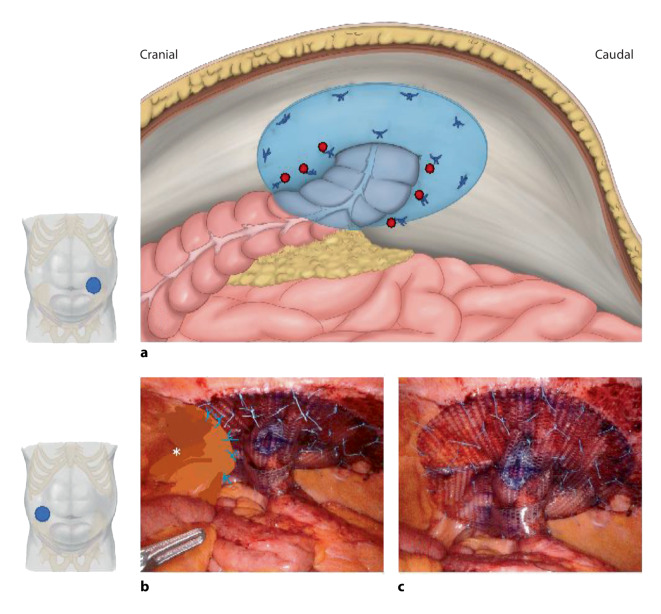

Ursprünglich wurde die Sugarbaker-Technik 1985 für die Laparotomie beschrieben und zielte darauf ab, die Darmaustrittstelle auf Ebene der Bauchdecke mit einem intraperitoneal platzierten Netz zu schließen, welches ohne Überlappung sozusagen als „Inlay“ in die Austrittstelle genäht wurde. Ab 2004 wurde die Technik modifiziert, indem das Netz deutlich vergrößert wurde, um eine zirkuläre Überlappung der Austrittstelle von 3–5 cm zu ermöglichen; dadurch entsteht ein Tunnel für den Durchtritt des Darmes (Abb. 1a; Video 1). Das Netz wurde nach wie vor in typischer IPOM(intraperitoneale Onlay-Mesh)-Position positioniert. Ab 2007 wurde der modifizierte Sugarbaker durch Filip Muysoms auch an die Laparoskopie angepasst [8]. Die roboterassistierte modifizierte Sugarbaker-Technik ist ergonomisch vorteilhafter als der laparoskopische Ansatz, da sie eine einfachere Visualisierung, Dissektion und Naht ermöglicht, der Faszienverschluss ist relativ einfach [17].

### Operationstechnik

Die Operation beginnt mit der Positionierung des ersten Ports im oberen Quadranten (subkostal) kontralateral zum Stoma. Zwei weitere Ports werden durch den Musculus transversus abdominis kaudal des ersten Ports platziert. Einer der Ports sollte 12 mm dick sein, um das Netz und die Nahtmaterialien einzuführen; die beiden weiteren Ports sind 8 mm dick. Die Ports werden mindestens eine Handbreit (7–8 cm) voneinander entfernt platziert, um die ungehinderte Funktion der Roboterarme zu gewährleisten. Netz und Nahtmaterialien können zu jedem Zeitpunkt nach Ende der Adhäsiolyse über den 12-mm-Port eingebracht werden. Das Netz wird entsprechend der Größe der Hernie(n) ausgewählt und dimensioniert, um eine ausreichende Überlappung zu gewährleisten. Bei grober Kontamination werden synthetische Netze vermieden. Mehrere nichtresorbierbare, geflochtene 0 USP-Nähte werden am Netz vorgelegt, um die Ausrichtung zu markieren und die Positionierung an der Bauchwand zu steuern. Das Netz wird aufgerollt und durch den 12-mm-Port eingebracht. In der Regel wird ein doppelt beschichtetes Polytetrafluorethylen(PTFE)-Netz verwendet, da es glatt ist und ein geringes Erosionsrisiko für den Darm aufweist.

Für eine optimale Synergie zwischen DaVinci Xi und Trumpf-Tisch ist eine Ausgangshöhe von 70 cm empfohlen. Unter direkter Sicht werden eine atraumatische Fasszange (z. B. Cadiere Forceps oder Fenestrated Bipolar Forceps) in den Port für die linke Hand und die monopolare Schere in den Port für die rechte Hand (bei Rechtshändern) eingeführt. Es erfolgt bei Bedarf die Adhäsiolyse des Omentums und Darmes mit der Schere, der Bruchsack wird nach intraabdominell mobilisiert. Um eine gute Überlappung des Netzes zu gewährleisten, wird anschließend ein präperitonealer Zugang zur Bauchdecke im inferolateralen Bereich geschaffen, beginnend am lateralen Rand der Bruchlücke. Diese Präparation ähnelt der präperitonealen Dissektion, die bei einer minimal-invasiven Leistenhernienversorgung als transabdominelle präperitoneale Patchplastik (TAPP) durchgeführt wird, und ermöglicht eine große Netzüberlappung nach dorsal und kaudal (Abb. 1b). Das Peritoneum dieser so gebildeten Tasche wird später verwendet, um einen Teil des Netzes zu bedecken; Fixationsnähte sollten nicht zu weit kaudal in Richtung Leistenkanal gesetzt werden, um eine Verletzung der neurovaskulären Strukturen zu vermeiden.

Die Stomaaustrittstelle wird in der Regel mit einer nichtresorbierbaren 0 UPS-Naht verschlossen (z. B. mit monodirektionalem Faden), entweder als Tabaksbeutelnaht oder als fortlaufende Naht; die Kalibrierung der Austrittstelle des Stomas durch die Bauchdecke muss den Darm gut, aber nicht zu fest umschließen. Der Darm wird zusammen mit dem zugehörigen Mesenterium mit extramukösen Nähten seitlich an die Bauchdecke fixiert, um seine Position in dem Tunnel der modifizierten Sugarbaker-Technik zu sichern. Das Netz wird schließlich mit den vorgelegten Nähten an die vordere Bauchdecke fixiert (transfasziale oder robotergestützte Naht): Begonnen wird mit der Platzierung der zentralen Naht am Rand der Stomaöffnung, dies ermöglicht die Zentrierung und Aufhängung des Netzes und erleichtert den gesamten Vorgang der Fixation. Der Sugarbaker-Tunnel entsteht, indem das Netz den Darm an die Bauchwand anschmiegt (Abb. 1a, rote Punkte). Seitliche Nähte werden an beiden Seiten des Tunnels angebracht. Es muss darauf geachtet werden, dass das Netz den Darm nicht zu eng umfängt (Cave: Ileus). Mit jeder Naht muss sichergestellt werden, dass weder Darm noch Mesenterium akzidentell mitgefasst werden. Die Nähte an der Peripherie des Netzes werden so gesetzt, dass das Netz straff an der Bauchwand anliegt (Abb. 1c). Die Peritonealtasche, die in der inferolateralen Position geschaffen wurde, wird abschließend über dem Netz befestigt, wobei auch hier darauf zu achten ist, dass neurovaskuläre oder Harnblasenverletzungen vermieden werden (Abb. 1b). Alle Nadeln und Operationshilfsmaterialien werden entfernt und auf Vollzähligkeit hin kontrolliert.

Häufigste Fehlerquellen beim modifizierten Sugarbaker sind die unzureichende Überlappung des Netzes (kurzer Tunnel mit weiter, trichterförmigen Öffnung, mit Rezidiv als Folge) oder aber die zu enge Befestigung des Netzes an die Bauchdecke (Passagehindernis und oder Schmerzen durch die Fixation). Wenn Darm oder Mesenterium redundant sind, besteht das Risiko der peristomalen Inkarzeration (inneren Hernie) oder des Volvolus [10, 18, 19].

### Ergebnisse und Kommentare

Die Ergebnisse dieses Operationsverfahrens wurden kürzlich veröffentlicht [17]. In einer Hochrisikogruppe von 15 Patienten wurden die Stomata bei den meisten Patienten aus onkologischen Gründen angelegt. 60 % der Patienten waren Raucher, hatten aber mindestens 4 Wochen vor der Operation mit dem Rauchen aufgehört. Bei einem Patienten kam es zu einer Enterotomie im Rahmen der Adhäsiolyse, bei diesem Patienten wurde alternativ ein biologisches Netz verwendet. Die mediane Aufenthaltsdauer betrug zwei Tage, was deutlich kürzer ist als bei Patienten nach offener oder laparoskopischer Versorgung [20, 21]. Die mittlere Operationszeit betrug 3 h. Postoperativ traten keine Wundkomplikationen auf, kein Patient musste innerhalb von 30 Tagen wieder aufgenommen werden. Bei einem Patienten (6,7 %) trat während der mittleren Nachbeobachtungszeit von 14,2 (± 9,4) Monaten ein Hernienrezidiv auf: Dieser Patient war morbid adipös (BMI 38) und hatte mehrere vorherige Versorgungen des Stomas mit Netz bekommen. Eine kritische Revision des Falles führte zur Weiterentwicklung der Technik im Sinne der Extraperitonealisierung des kaudalen Netzanteils, um eine größere untere Netzüberlappung zu gewährleisten.

## Die Pauli-Technik

Im Jahr 2016 beschrieben Pauli et al. ein Verfahren zur retromuskulären parastomalen Hernienversorgung, bei der ein Transversus-abdominis-Release (TAR) mit den Vorteilen des Tunnels der modifizierten Sugarbaker-Technik sowie der Extraperitonealisierung des Netzes kombiniert werden (Video 2; [10]). Obwohl ursprünglich als offene Technik beschrieben, sind inzwischen auch laparoskopische und roboterassistierte Eingriffe publiziert worden [22, 23].

### Operationstechnik

Üblich ist der laparoskopische transabdominelle Zugang zur sicheren Reduktion des Hernieninhalts. Begonnen wird mit dem Einstieg in den Retrorektusraum, durch Eröffnung der hinteren Rektusscheide nahe an der Linea alba, auf der ipsilateralen Seite des Stomas, im Sinne eines einseitigen TAR. Wenn der mediale Abstand vom Stoma zur Linea alba für eine ausreichende Überlappung des Netzes nach kontralateral nicht ausreicht, kann der Zugang über die kontralaterale Rektusscheide im Sinne eines TARUP („transabdominal retrorectus umbilical prosthesis“) mit Crossover hinter der Linea alba und dann Fortführung als TAR durchgeführt werden [12, 24]. Der Eingriff kann alternativ total extraperitoneal durchgeführt werden („enhanced-view totally extraperitoneal plasty“, eTEP), auch bei konkomitanter Reparation von Hernien der Linea alba. Wenn ein beidseitiger TAR erforderlich ist, ist in der Regel das Zweifache Andocken (auf jeder Seite, respektive), ähnlich wie für den klassischen roboterassistierten TAR (r-TAR) beschrieben, erforderlich [13].

**Figure 2:**
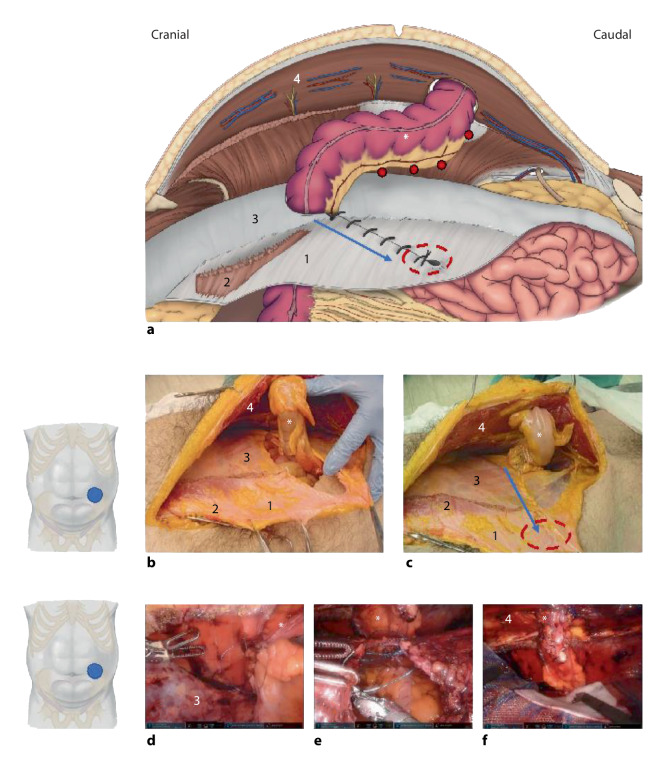

Nach Dissektion und Freilegung des Retrorektalraums auf eine Fläche, die 10 cm kranial und kaudal des Stomas reicht, wird der Bruchsackhals ringsum inzidiert bzw. abgelöst. Anschließend folgt die TAR-Dissektion lateral des Stomas; der TAR kann kranial (top-down) oder kaudal vom Bogros-Raum aus („bottom-up“) begonnen werden. Ausgehend vom Bogros-Raum wird die Dissektionsebene typischerweise dorsal der Fascia transversalis entwickelt. Das Peritoneum ist im kranialen Teil der vorderen Bauchwand sehr dünn [13]. Die Fascia endoabdominalis und der M. transversus abdominis werden medial zu den neurovaskulären Bündeln eingeschnitten und der Raum nach lateral weiter präpariert, mindestens aber 10 cm lateral der Stomaaustrittstelle und in der Regel nicht weniger als 18 cm in kraniokaudaler Ausdehnung. Nach der Durchführung des TAR und Schaffung einer entsprechenden „Landezone“ für das Netz wird die hintere retromuskuläre Faszie lateral der Stomaaustrittstelle bis an die Grenze der präparierten TAR-Ebene eingeschnitten (Abb. 2a). Anschließend wird der zum Stoma führende Darm so positioniert, dass er ohne Redundanzen lateralisiert wird (ähnlich wie bei dem modifizierten Sugarbaker, allerdings zwischen den Bauchdeckenschichten; Abb. 2a–c).

Der Darm wird mit einem 23 cm langen resorbierbaren monodirektionalen 2‑0 USP-Nahtmaterial an die Bauchdecke fixiert, das Mesenterium wird auch von lateral nach medial auf einer Länge von 6–7 cm auf der kranialen Seite fixiert, ohne die Gefäßversorgung zu kompromittieren (Abb. 2d). Es muss darauf geachtet werden, dass einerseits das Stoma nicht auf Hautniveau eingezogen wird und andererseits keine Längenredundanz des austretenden Darmes zwischen den Bauchdeckenschichten entsteht. Die Netzfixation ist in den meisten Fällen überflüssig. Die peritoneoaponeurotische Schicht wird, beginnend an der lateralisierten „inneren“ Stomaaustrittstelle, von lateral nach medial verschlossen (Abb. 2a, c: *blauer Pfeil*; Abb. 2e). Es ist darauf zu achten, dass die neue innere Öffnung den Dram nicht einschnürt. Die innere Lateralisierung der Stomaaustrittstelle sollte einen Abstand von mindestens 8 cm zur äußeren Austrittstelle betragen. Je nach Befund kann die eigentliche Stomaaustrittstelle mit einer nichtresorbierbaren Naht etwas eingeengt und an den durchtretenden Darm angepasst werden.

Der Raum für die Netzpositionierung wird mit dem Lineal ausgemessen. Die Größe des Netzes sollte erfahrungsgemäß mindestens 18 × 18 cm betragen. Das verwendete Netz sollte großporig, synthetisch und nichtresorbierbar sein; üblich sind Netze aus Polypropylen, PVDF (Polyvinylidenfluorid), expandiertem PTFE (ePTFE) oder Polyester. Zur Vorbeugung der Erosion des Netzes in den Darm und das Mesenterium (mit Spätkomplikationen wie Devaskularisierung, Infektion und Fistelbildung) kann bei großporigen Netzen ein zusätzliches resorbierbares Netz als Schutzschicht verwendet werden („scaffold“; Abb. 2f). Bei erhöhtem Risiko für Darmfisteln wie beim M. Crohn gibt es keine optimale Lösung; die Verwendung eines resorbierbaren biosynthetischen Netzes als Schutzschicht oder alternativ ein mikroporöses ePTFE-Netz können in einzelnen Fällen von Vorteil sein. Grundsätzlich muss das Netz in dieser Position (wie vergleichsweise auch beim klassischen TAR) nicht fixiert werden. Da der Darm an die Bauchdecke fixiert ist und das Netz nicht fixiert wird, kann sich das Netz spannungsfrei an den Darm anpassen, was wiederum das Risiko einer Darmeinengung verringert. Bei transabdominellem Zugang wird abschließend die retromuskuläre Tasche mit fortlaufender Naht verschlossen.

### Ergebnisse und Kommentare

Die Autoren haben das Pauli-Verfahren auf die Laparoskopie übertragen und verfügen über die Erfahrung an 26 roboterassistierten Pauli-Eingriffen mit einer Nachbeobachtungszeit von im Median 14 Monaten (Range: 0–30). Die Patienten hatten ein Durchschnittsalter von 64 ± 8 Jahren, 14 waren männlich (54 %), der mittlere BMI lag bei 27 (Range: 21–36), 5 Patienten waren Raucher mit COPD („chronic obstructive pulmonary disease“) und 4 hatten Diabetes mellitus. Die mediane Zeit des Vorhandenseins des Stomas vor der Operation betrug 48 Monate (Range 12–251), 3 Patienten hatten bei der Indexoperation ein prophylaktisches Netz bekommen, bei 8 Patienten (31 %) handelte es sich um ein Rezidiv der parastomalen Hernie (*n* = 4 nach Netzverfahren; *n* = 3 Rerezidive). Die meisten Patienten hatten das Stoma wegen eines Rektumkarzinoms (13 Patienten); andere Ursachen waren Anal- oder Harninkontinenz (3 querschnittgelähmte Patienten). Die Stomata waren allesamt endständig, 20 Kolostomata, 5 Ileostomata und eine Urostoma. Bei 6 Patienten wurde eine konkomitante mediane Inzisionalhernie mittels bilateralem r‑TAR versorgt, bei 5 Patienten wurde der eTEP-Zugang gewählt. Bei 3 Patienten musste in gleicher Sitzung eine Stomarevision durchgeführt werden, 2 davon waren geplante Eingriffe; bei 7 Patienten traten Serosaläsionen auf, und bei einem Patienten führte eine Vollwandläsion auf Hautniveau zur Notwendigkeit der In-situ-Relokation des Stomas. Die mittlere Operationszeit betrug 156 min für r‑Pauli allein (Range: 107–233) und 265 min für r‑Pauli mit konkomitanter Inzisionalhernienversorgung und/oder zusätzlicher Stomarevision (Range: 160–314).

Postoperative Komplikationen traten bei 8 Patienten auf (31 %). Bei einem Patienten kam es zu einer Stomanekrose und einer subkutanen Infektion des Darmes, der 3 Wochen nach der Indexoperation revidiert und das Stoma reloziert wurde. Drei Patienten hatten einen postoperativen Ileus, 3 Patienten hatten akute Schmerzen und bei einem Patienten wurde wegen Schmerzen ohne pathologischen Befund eine Second-look-Laparoskopie durchgeführt. Vier Patienten entwickelten ein Flankenhämatom oder Serom, das bei 3 Patienten spontan zurückging; bei einem Patienten wurde das Serom mittels Drainageneinlage behandelt. Kein Patient berichtete nach 30 Tagen über chronische Schmerzen. Die mediane Dauer des postoperativen Aufenthalts betrug 3 Tage (Range: 1–13). Ein Patient mit Ileus und Nierenversagen und ein Patient mit Serom mussten erneut stationär aufgenommen werden. Bei komplikationslosem r‑Pauli und einer medianen Nachbeobachtungszeit von 14 Monaten trat kein Rezidiv auf; der Patient, bei dem die Stomanekrose aufgetreten war und bei dem eine Neuanlage mit Relokation erfolgte, entwickelte eine Inzisionalhernie im Bereich des ursprünglichen Stomas (3,8 % oder 1/26).

## Die IPST-Technik mit trichterförmigem Netz

**Figure 3:**
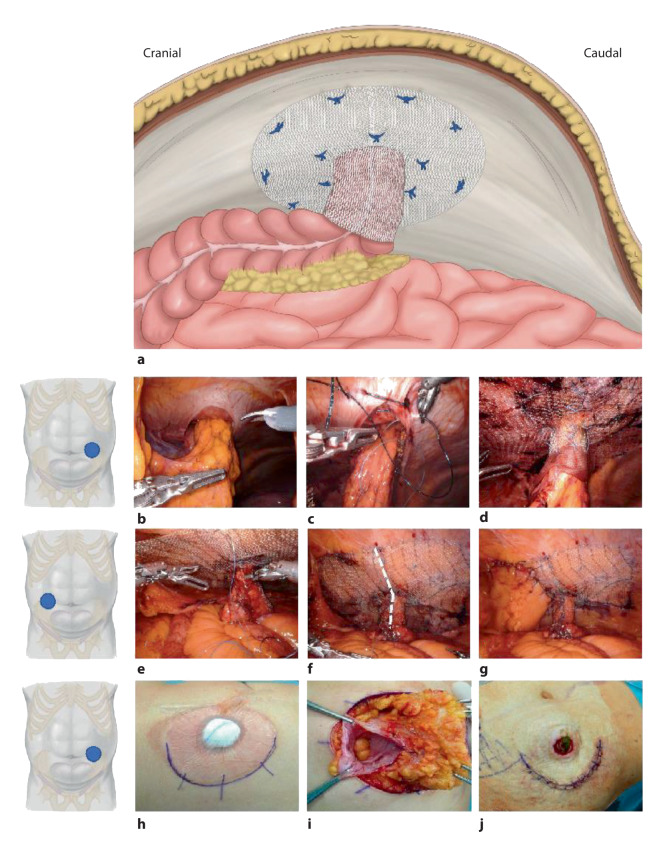

Für die Versorgung und Prophylaxe der parastomalen Hernie wurde 2008 ein neues Netz entwickelt, das die Eigenschaften eines Trichters (zur Verhinderung von Teleskopierung und Prolaps) und eines angepassten Rollkragens (zur Verhinderung eines Rezidivs) kombiniert. Das IPST-Netz besteht aus reinem PVDF auf der viszeralen Seite und Polypropylen/PVDF auf der parietalen Seite und enthält Eisenpartikel in der Polymerstruktur, die bei Bedarf eine Bildgebung mittels Magnetresonanztomographie ermöglichen (Dynamesh, Aachen, Deutschland). Der Trichter ist 4 cm lang und hat einen Durchmesser von 2,5 cm; da er gestrickt ist und eine gewisse Plastizität aufweist, kann der Durchmesser zur besseren Akkommodation von Darm und Mesenterium digital erweitert werden. Das Netz wird in IPOM-Position implantiert ([25]; Abb. 3a; Video 3). Es gibt geschlitzte (für die Anwendung ohne Stomarelokation) und nichtgeschlitzte IPST-Netze (für die prophylaktische Implantation oder bei Stomarelokation).

### Operationstechnik

Die Trokarpositionierung wird wie oben beschrieben kontralateral zur Seite des Stomas durchgeführt. Nach der Adhäsiolyse und Reposition der Hernie wird das geschlitzte IPST-Netz über den 12-mm-Port eingeführt, um die Stomaschlinge gelegt und der Trichter mit unterbrochenen, nichtresorbierbaren Nähten verschlossen (Abb. 3e, f). Anschließend wird die flache Oberfläche des Netzes an die Bauchdecke positioniert und auch dieser Teil des Schlitzes mit Nähten verschlossen (Abb. 3e, f). Wie oben für die modifizierte Sugarbaker-Technik beschrieben, ist es unter bestimmten Umständen sinnvoll, einen Teil der Leistenregion zu parietalisieren, um eine sichere „Landezone“ für die Netzplatzierung bzw. eine optimale Netzinkorportion zu ermöglichen. Dieser Peritoneallappen bedeckt den kaudalen Teil des Netzes und verringert das Risiko von Adhäsionen am Netz (Abb. 3g). Die Stomaaustrittstelle wird, wie auch oben für die anderen Techniken beschrieben, mit Naht auf den idealen Durchmesser eingeengt (Abb. 3b, c/Kolostoma). Die Fixation des Netzes erfolgt mit nichtresorbierbarem Nahtmaterial in Double-crown-Technik (Abb. 3a, d).

In Fällen, bei denen der Bruchsack, der Dick- oder Dünndarm, das Mesenterium und das Omentum fest siphonartig aneinanderhaften und über einen intraabdominalen Zugang nur schwer adhäsiolysierbar sind, kann in einem Hybridverfahren ein Zugang über einen peristomalen Schnitt geschaffen werden (Abb. 3h–j). Wenn das Stoma komplett aus der Haut gelöst wird, kann ein ungeschlitztes IPST-Netz verwendet und von außen eingelegt werden; die korrekte Entfaltung und Fixierung des Netzes erfolgt dann in der zweiten anschließenden laparoskopisch-robotischen Phase der Operation. Vor dem erneuten Andocken wird die Stomaaustrittstelle mit nichtresorbierbaren Nähten getrimmt. Das neu implantierte Stoma verbleibt somit in der gleichen Position, ein Verfahren das als „In-situ“-Relokation bekannt ist [9].

### Kommentare

Dieses Verfahren wurde von den Autoren an 6 Patienten roboterassistiert durchgeführt, einmal bei parastomaler Hernie eines Ileum-Conduits, zweimal bei endständigem Ileostoma nach Proktokolektomie und dreimal bei endständigem Kolostoma. Die mittlere Operationszeit betrug 201 min (Range: 129–241); die Operationszeit ist maßgeblich von dem Aufwand für die Adhäsiolyse abhängig.

Bei einem der Patienten mit Ileostomie und einem BMI von 32 war am 5. postoperativen Tag eine Revision wegen eines Ileus erforderlich. Bei der Revision zeigten sich Verwachsungen am IPST-Trichter, der Trichterschlitz wurde eröffnet und die Reparation de facto in ein Keyhole-Verfahren umgewandelt; wie zu erwarten, entwickelte dieser Patient anschließend ein Rezidiv und wurde ergänzend mit roboterassistiertem modifiziertem Sugarbaker (Sandwich-Technik) versorgt (Abb. 1c). Bei 3 von 6 Patienten wurde ein hybrider Ansatz mit subkutaner peristomaler Adhäsiolyse durchgeführt. Der Patient mit IPST-Netz am Ileum-Conduit ist bereits seit 4 Jahren rezidivfrei und sehr zufrieden. Neben diesen 6 roboterassistierten Fällen, verfügen die Autoren über Erfahrung an mehr als 100 Patienten nichtroboterassistierter endoskopischer IPST-Netzimplantationen zur Prävention und Reparation parastomaler Hernien [26, 27].

Die IPST-Technik bietet mehrere Vorteile: Das Verfahren ist hochgradig standardisiert, erlaubt dank der Elastizität des dehnbaren Trichters eine sichere kragenförmige Umhüllung des austretenden Darms, es können begleitende Narbenhernien mitversorgt werden, die Neigung des Stomas, zu prolabieren, ist gering, und die seitliche Schwachstelle des modifizierten Sugarbaker-Verfahrens ist nicht vorhanden. Nicht zuletzt erleichtert die Trichterkonfiguration, bei der der Darm in einem 90°-Winkel durch die Bauchdecke geführt wird, die Irrigation des Stomas: Diese kann durch die Lateralisierung des Darms bei der modifizierten Sugarbaker-Technik und dem r‑Pauli-Verfahren unter Umständen beeinträchtigt sein.

## Versorgung der parastomalen Hernie am Ileum-Conduit

Im Vergleich zu Ileo- oder Kolostomata haben parastomale Hernien am Ileum-Conduit einige zusätzliche Besonderheiten, welche die Versorgung erschweren (Video 4). Im Allgemeinen sind die angewandten Techniken für die chirurgische Behandlung von Ileum-Conduit-Hernien ähnlich wie die für andere Hernien. Je nach intraoperativem Befund ist allerdings ein individuell maßgeschneiderter Ansatz nötig. Dies impliziert ein breites chirurgisches Wissen, bei dem roboterassistierte Techniken im Vordergrund stehen. Die besonderen Herausforderungen bei der Behandlung parastomaler Hernien am Ileum-Conduit wurden in einer kürzlich veröffentlichten technischen Mitteilung beschrieben und werden im Folgenden zusammengefasst [28].

**Figure 4:**
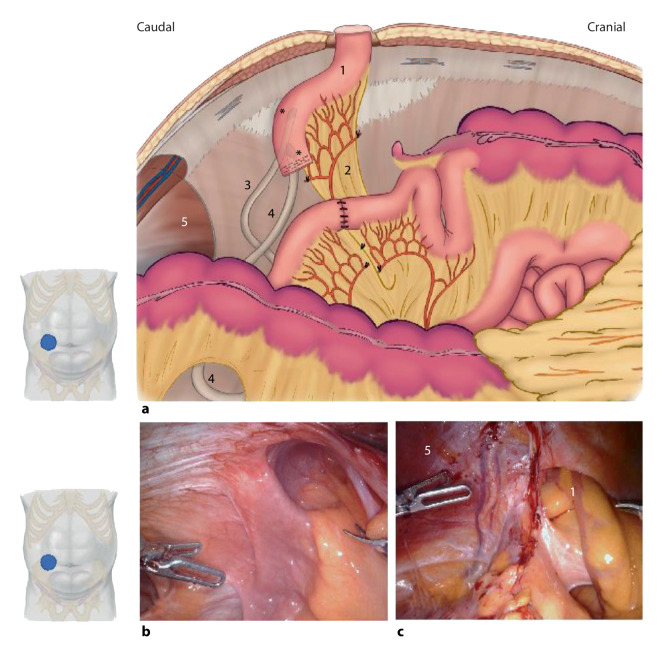

Erstens beinhaltet eine radikale Zystektomie häufig die Entfernung des Peritoneums und des präperitonealen Fettgewebes unterhalb der Linea arcuata. Dies macht unter Umständen den extraperitonealen Zugang unmöglich, sodass auf den modifizierten Sugarbaker, ein Keyhole-Netz oder ein IPST-Netz (alle drei in IPOM-Position) ausgewichen werden muss. Der Zustand nach Peritonektomie erschwert auch den Verschluss der hinteren Schicht im Rahmen des TAR bei der konkomitanten Mitversorgung von Inzisionshernien der Mittellinie. In solchen Fällen sind Überbrückungstechniken mit Omentum oder resorbierbarem Netz sinnvoll. Zweitens, wird die Dissektion des Ileum-Conduits durch die oft langjährig geringe Füllung des Conduits und das Vorhandensein der Ureteranastomosen in unmittelbarer Nähe oft erschwert. Die spezifische anatomische Situation nach einer radikalen Zystektomie mit Harnableitung über ein Ileum-Conduit ist in Abb. 4 dargestellt. Eine weitere Beobachtung, die bei parastomalen Hernien am Ileum-Conduit gemacht wird, ist die schwierige Lateralisierung der Stomaschlinge: Wegen des oft kurzen Mesenteriums ermöglichen intraperitoneale oder retromuskuläre modifizierte Sugarbaker-Reparationen oft eine nur unzureichende Netzüberlappung. Für solche Fälle sind ein Keyhole-Netz oder das IPST-Netz sinnvoll.

### „Tailored approach“

**Figure 5:**
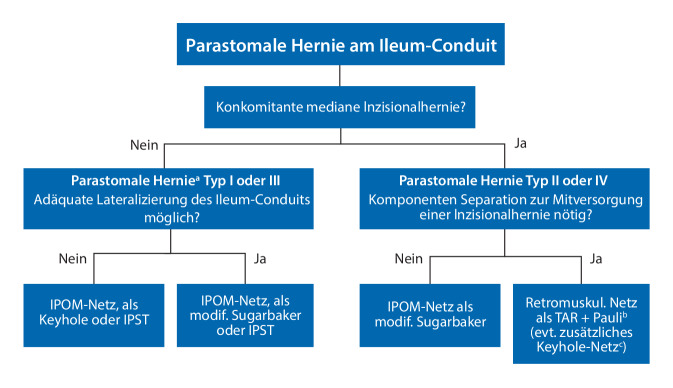

Bei fast der Hälfte der Patienten mit parastomaler Hernie am Ileum-Conduit besteht gleichzeitig eine Inzisionshernie der Mittellinie [29]. Obwohl zunehmend minimal-invasive Techniken zur Durchführung der radikalen Zystektomie eingesetzt werden, wird die Mehrzahl der Fälle offen operiert [30]. Zusammen mit den oben beschriebenen Herausforderungen unterstreicht dies die Notwendigkeit guter strategischer Planung bei der Versorgung parastomaler Hernien am Ileum-Conduit. Wenn eine konkomitante mediane Inzisionalhernie eine Komponentenseparation erfordert, bietet der roboterassistierte Ansatz einzigartige Vorteile, der r‑TAR kann in Kombination mit dem r‑Pauli durchgeführt werden [10]. Die perioperative retrograde Füllung des Ileum-Conduits mit indigocarminblau gefärbter Kochsalzlösung über einen Foley-Katheter ist sowohl bei der intraoperativen Identifizierung der Stomaschlinge als auch bei der Erkennung perioperativer Deserosierungen oder transmuraler Läsionen von großer Hilfe. Das Flussdiagramm zeigt einen möglichen „tailored approach“ für die roboterassistierte Versorgung parastomaler Ileum-Conduit-Hernien (Abb. 5).

### Ergebnisse und Kommentare

Eine erste Erfahrung mit diesem „tailored approach“ wurde kürzlich veröffentlicht [28]. In einem Zeitraum von 4 Jahren wurde bei 15 Patienten die minimal-invasive Versorgung einer parastomalen Hernia an einem Ileum-Conduit durchgeführt. Bei fast der Hälfte der Patienten (7/15, 46,7 %) lag gleichzeitig eine Inzisionshernie in der Mittellinie vor. Die Mehrzahl der Patienten wurde roboterassistiert operiert (10/15; 66,7 %). Der Eingriff dauerte im Median 197 min (Range: 132–260). Bei 33,3 % der Patienten (5/15) wurde das Netz extraperitoneal positioniert, während die übrigen Patienten mit einem intraperitonealen Netz operiert wurden. In einem Fall wurde die roboterassistierte Operation aufgrund einer perioperativen Laesio bei der Adhäsiolyse am Ileum-Conduit in eine offene Operation umgewandelt. Der mediane postoperative Krankenhausaufenthalt betrug 5 Tage. Bei fast der Hälfte der Patienten (7/15, 46,7 %) trat innerhalb von 30 Tagen eine Komplikation auf: 33 % bekamen eine Harnwegsinfektion, 2 Patienten mussten während des Krankenhausaufenthalts intensivmedizinisch betreut werden. Die mediane Nachbeobachtungszeit betrug 366 Tage. Eine Patientin entwickelte am 66. postoperativen Tag ein lokales Rezidiv ihrer parastomalen Hernie, das mit einem intraperitonealen Netz behandelt wurde. Am Beispiel dieser Serie mit 15 Patienten wird deutlich, dass die Versorgung parastomaler Hernien am Ileum-Conduit eine komplikationsträchtige Herausforderung bleibt.

Über die chirurgische Behandlung parastomaler Hernien bei Ileum-Conduit gibt es nur wenige Daten [11, 29–31]. Bisher wurden nur retrospektive Patientenserien veröffentlicht, die eine begrenzte Anzahl von Patienten umfassten. Kürzlich zeigte auch eine Analyse aus Finnland die erhebliche Morbidität der Operation [22]: Retrospektiv wurden 28 Patienten analysiert, die zwischen 2007 und 2017 mit verschiedenen Techniken behandelt worden waren. Während einer medianen Nachbeobachtungszeit von 30 Monaten wurden 18 % Rezidive und 14 % Komplikationen festgestellt. Es wurde über leicht bessere Ergebnisse der modifizierten Sugarbaker-Technik im Vergleich zum Keyhole-Netz berichtet. Die verfügbare Literatur erlaubt keinen Schluss über die optimale chirurgische Therapie parastomaler Hernien am Ileum-Conduit. Neue Entwicklungen, wie roboterassistierte Techniken, die konkomitante Mitversorgung medianer Inzisionalhernien mittels r‑TAR oder die Reparation parastomaler Hernien in der r‑Pauli-Technik, sind in keiner der verfügbaren Kohortendaten enthalten. Nicht zuletzt besteht zwar Einigkeit darüber, dass die Anpassung der Operationsart an die individuellen Merkmale des Patienten und den Befund der parastomalen Hernie am Ileum-Conduit von größter Bedeutung sind, doch wird wegen der Seltenheit dieser Befunde die Datenlage auch in Zukunft nicht viel besser sein; wahrscheinlich macht es Sinn, dass diese Operationen zentralisiert oder zumindest die Daten in prospektive Register aufgenommen werden.

## Fazit für die Praxis

- Parastomale Hernien beeinträchtigen die Lebensqualität erheblich.
- Die chirurgische Versorgung parastomaler Hernien ist komplex, Komplikationen und Rezidive sind häufig.
- Anhand der derzeitigen Datenlage kann keine Aussage über die optimale Operationstechnik getroffen werden.
- Die chirurgische Versorgung parastomaler Hernien wird zunehmend minimal-invasiv durchgeführt. Die roboterassistierte Operation hat spezifische Vorteile, wie die Vermeidung penetrierender oder transfaszialer Fixationstechniken, den einfachen Nahtverschluss der Stomaaustrittstelle, die Erleichterung der extraperitonealen Netzpositionierung und die Anwendung komplexer Komponentenseparationstechniken.
- Das Operationsverfahren zur Versorgung parastomaler Hernien sollte auf die Anamnese des Patienten, die Merkmale der Hernie und das Vorhandensein einer begleitenden Inzisionalhernie maßgeschneidert erfolgen.

## Supplementary Information







